# Carbohydrates in plant immunity and plant protection: roles and potential application as foliar sprays

**DOI:** 10.3389/fpls.2014.00592

**Published:** 2014-11-04

**Authors:** Sophie Trouvelot, Marie-Claire Héloir, Benoît Poinssot, Adrien Gauthier, Franck Paris, Christelle Guillier, Maud Combier, Lucie Trdá, Xavier Daire, Marielle Adrian

**Affiliations:** ^1^Université de Bourgogne, UMR AgroSup/INRA/uB 1347 Agroécologie, Pôle Interactions Plantes-Microorganismes-ERL CNRS 6300Dijon, France; ^2^Department of Biosciences, Plant Biology, University of HelsinkiHelsinki, Finland; ^3^INRA, UMR AgroSup/INRA/uB 1347 Agroécologie, Pôle Interactions Plantes-Microorganismes-ERL CNRS 6300Dijon, France

**Keywords:** carbohydrates, oligosaccharides, sugars, immunity, plant defense, signaling, elicitor, phyllosphere microflora

## Abstract

Increasing interest is devoted to carbohydrates for their roles in plant immunity. Some of them are elicitors of plant defenses whereas other ones act as signaling molecules in a manner similar to phytohormones. This review first describes the main classes of carbohydrates associated to plant immunity, their role and mode of action. More precisely, the state of the art about perception of “PAMP, MAMP, and DAMP (Pathogen-, Microbe-, Damage-Associated Molecular Patterns) type” oligosaccharides is presented and examples of induced defense events are provided. A particular attention is paid to the structure/activity relationships of these compounds. The role of sugars as signaling molecules, especially in plant microbe interactions, is also presented. Secondly, the potentialities and limits of foliar sprays of carbohydrates to stimulate plant immunity for crop protection against diseases are discussed, with focus on the roles of the leaf cuticle and phyllosphere microflora.

## Introduction

Plants possess an immune system that allows defending themselves against a wide range of microorganisms including bacteria, oomycetes and fungi (Gomez-Gomez and Boller, 2000; Nürnberger et al., 2004; Zipfel and Felix, 2005; Boller and Felix, 2009). Activation of defense reactions implies the essential step of the microorganism detection by highly conserved molecular patterns called PAMPs (Pathogen Associated Molecular Patterns) or MAMPs (Microbe Associated Molecular Patterns) (Nürnberger et al., 2004; Chisholm et al., 2006; Jones and Dangl, 2006; Boller and Felix, 2009) which are secreted by microorganisms or released from their cell wall by hydrolytic enzymes during interaction with the plant. Their perception during pathogen infection triggers defense reactions known as PAMP-triggered immunity (PTI) (Jones and Dangl, 2006). So they are considered as general elicitors i.e., compounds able to induce plant defenses (Ebel and Cosio, 1994). These general elicitors can also derive from the plant cell walls during plant microbe interactions after hydrolysis by pathogen cell wall degrading enzymes (Vidal et al., 1998; Boudart et al., 2003) and are therefore called DAMPs (Damage-Associated Molecular Patterns). General elicitors belong to various biochemical classes including carbohydrates, lipids, (glyco)peptides and (glyco)proteins. In this paper, attention is paid to “PAMP, MAMP and DAMP type” carbohydrates, their perception by plants and the induced defense events.

In plants, carbohydrates produced by photosynthesis are well known for their essential role as vital sources of energy and carbon skeletons for organic compounds and storage components. Additionally, a pivotal function as signaling molecules, in a manner similar to hormones, has become apparent (Koch, 1996, 2004; Sheen et al., 1999; Rolland et al., 2006; Smeekens et al., 2010) and is nowadays largely investigated. Hence, as they interact with diurnal changes, abiotic and biotic stresses, and hormone signaling, sugars are considered as actors of a complex communication system necessary for the coordination of metabolism with growth, development, and responses to environmental changes and stresses (Rolland et al., 2002, 2006). Sugars, especially the disaccharides sucrose and trehalose, raffinose family oligosaccharides and fructans also play a role regarding ROS produced by plants in response to abiotic stresses. Known plants antioxidants are enzymatic scavengers (superoxide dismutase, ascorbate peroxidase, glutathione peroxidase) and non-enzymatic metabolites (ascorbate, glutathione, α-tocopherol). In addition, there is growing evidence for a role of sugars as antioxidants as they possess ROS scavenging properties. Sugars could therefore be considered as key components of an integrated cellular redox network. As this role was recently reviewed in details by Keunen et al. (2013), it was not developed here.

In plant microbe interactions, sugars are essential to fuel the energy required for defenses and serve as signals for the regulation of defense genes (Ehness et al., 1997; Roitsch et al., 2003; Bolton, 2009). The potential key roles of some sugars regarding plant immunity have recently led to the “sweet Immunity” and “sugar-enhanced defense” concepts (Bolouri-Moghaddam and Van Den Ende, 2013).

Regarding their roles in plant immunity, the question is to determine whether carbohydrates could be helpful in controlling plant diseases in field conditions (Delaunois et al., 2014). Elicitor-induced resistance against pathogens is a strategy of crop protection under investigation (Walters et al., 2013). Hence, pesticides remain largely used to prevent crops from diseases but the secondary effects of some of them regarding the environmental quality, human health, and selection of resistant strains stimulate research for the development of new strategies in a context of sustainable crop production. Various carbohydrates are presently studied and experimented for their possible role as resistance inducers. Among the biggest challenges of this strategy are their low level of penetration through the cuticle, which limits their perception, and their possible alteration and/or metabolism by microorganisms of the phyllosphere.

## Carbohydrates and plant immunity

### Main classes of carbohydrates involved in plant immunity

Mono- and disaccharides such as glucose, sucrose or trehalose are the smallest carbohydrates, generally referred as sugars. Oligo- and polysaccharides are naturally occurring complex carbohydrates formed by chains of sugar residues interconnected by glycosidic linkages and with biological regulatory functions (Albersheim et al., 1983). Different structural patterns have been reported and described for oligo-and polysaccharides, including β-glucans, chitin- and chitosan oligomers, oligogalacturonides, alginates, fucans, carrageenans, and ulvans (Côté and Hahn, 1994; Klarzynski et al., 2000, 2003) (Table 1).

**Table 1:** **Structures of the main di- and oligosaccharides reported as elicitors of plant defenses and/or resistance inducers against pathogens**.

**Carbohydrate**	**Structure or repetitive units**	**References reporting induction of defenses and/or plant resistance**
Sucrose	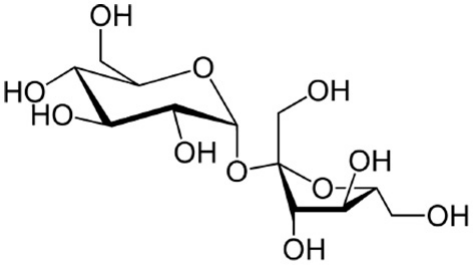	Rolland et al., 2002; Morkunas et al., 2005; Gómez-Ariza et al., 2007; Wind et al., 2010; Bolouri-Moghaddam and Van Den Ende, 2012, 2013
Trehalose	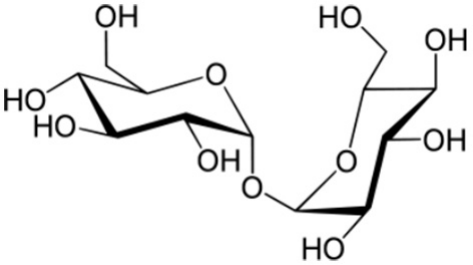	Reignault et al., 2001; Muchembled et al., 2006; Renard-Merlier et al., 2007; Fernandez et al., 2010; Singh et al., 2011
β-1,3 glucans: example of laminarin	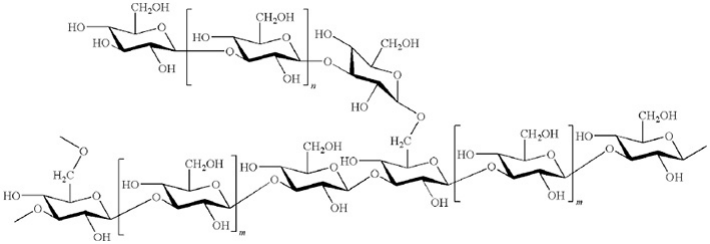	Kobayashi et al., 1993; Inui et al., 1997; Cardinale et al., 2000; Klarzynski et al., 2000; Aziz et al., 2003; Renard-Merlier et al., 2007; Fu et al., 2011; Gauthier et al., 2014
Sulfated β-1,3 glucans Example of the sulfated laminarin PS3 (*Phycarin Sulfated 3)*	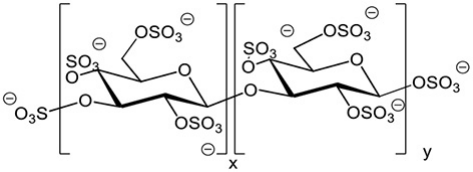	Ménard et al., 2004; Ghannam et al., 2005; Ménard et al., 2005; Trouvelot et al., 2008; Steimetz et al., 2012; Gauthier et al., 2014
Fucans	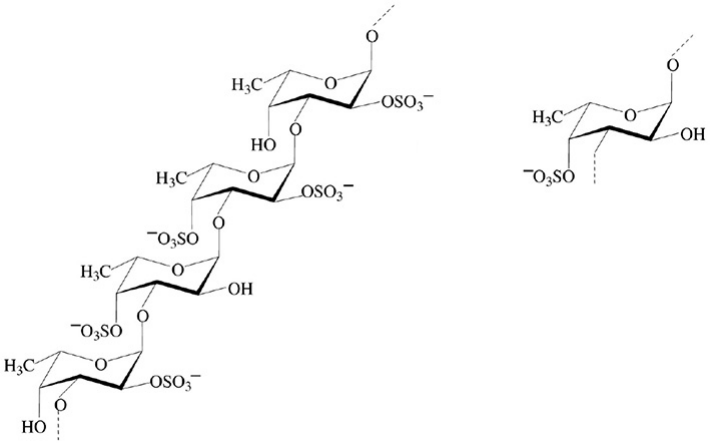	Lizzi et al., 1998; Klarzynski et al., 2003
Carrageenans	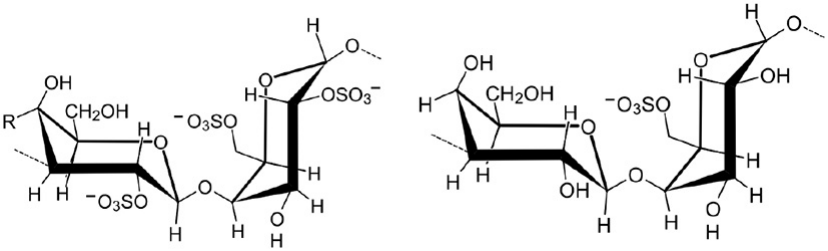	Patier et al., 1995; Bouarab et al., 1999; Mercier et al., 2001; Sangha et al., 2010; Vera et al., 2012
Ulvans	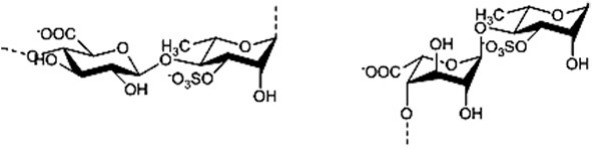	Cluzet et al., 2004; Abreu et al., 2008; Araujo et al., 2008; Borsato et al., 2010; Jaulneau et al., 2010, 2011; Freitas and Stadnik, 2012; Araújo and Stadnik, 2013; Delgado et al., 2013; Stadnik and De Freitas, 2014
Alginates (G blocks: poly D-glucuronic acid) (M blocks: poly D-mannuronic acid) (GM blocks: alternate D-glucuronic and D-mannuronic acid)	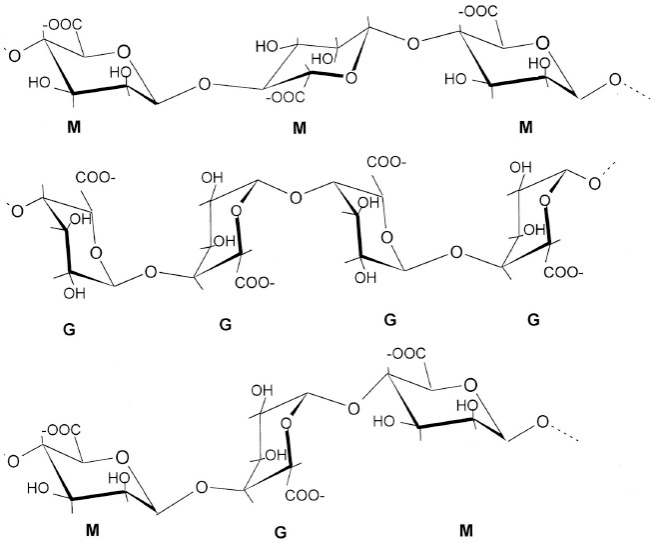	Potin et al., 1999; Akimoto et al., 2000; Chandía et al., 2004; An et al., 2009
Chitin	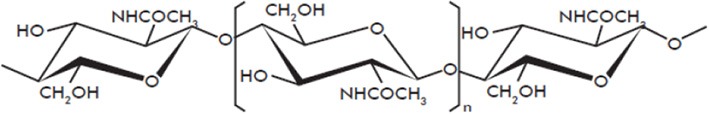	Pearce and Ride, 1982; Kuchitsu et al., 1993; Kaku et al., 2006; Eckardt, 2008; Hamel and Beaudoin, 2010; Sharp, 2013
Chitosan	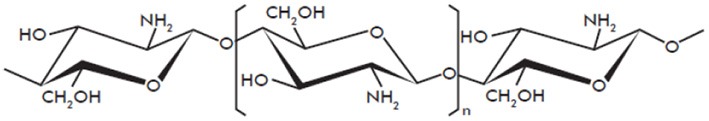	Kohle et al., 1985; Doares et al., 1995; Lafontaine and Benhamou, 1996; Vasyukova et al., 2001; Cabrera et al., 2006; Amborabe et al., 2008; Iriti and Faoro, 2009; Vasil'ev et al., 2009; Cabrera et al., 2010; El Hadrami et al., 2010; Hamel and Beaudoin, 2010; Li et al., 2014
Oligogalacturonides	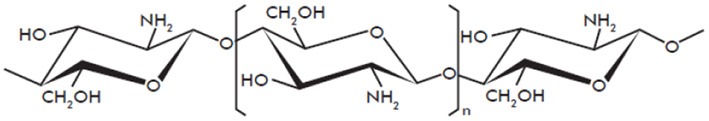	Hahn et al., 1981; Davis et al., 1986; Davis and Hahlbrock, 1987; Cabrera et al., 2008, 2010; Galletti et al., 2008; Rasul et al., 2012; Ferrari et al., 2013

Beta-glucans are ubiquitous in plant and fungal cell walls. The β-1,4 glucan cellulose is one of the most abundant glucans in plants. Among β-1,3 glucans, laminarin, a storage polysaccharide from the brown algae *Laminaria digitata*, has an average degree of polymerization (DP) of 25–33 glucose units with up to three single β-glucose branches at position 6 (Read et al., 1996; Lepagnol-Descamps et al., 1998; Klarzynski et al., 2000).

Chitin is the second most ubiquitous natural polysaccharide after cellulose. It is not a pure homopolymer but rather an heteropolymer of β-1,4-linked *N*-acetylglucosamine with a varying percentage of deacetylated glucosamine (Merzendorfer, 2011). Chitin is a major component of fungal cell walls and is also present in the cuticle of non-vertebrates such crustacean shells, insect exoskeletons, and in eggs of parasitic nematodes, protists, algae (Bueter et al., 2013). Chitosan, the deacetylated derivative of chitin produced by chitin deacetylases, is a less common natural polysaccharide. It is notably found in zygomycete cell walls (Mohammadi et al., 2012).

Pectin is the most complex plant cell wall polysaccharide due to the numerous sugar monomers and types of linkages involved in the branched rhamnogalacturonans I and II domains, and to the variable level of esterification of the homogalacturonan domain. Oligogalacturonides (OGAs) are linear molecules composed of oligomers of α-1,4-linked galacturonosyl residues more or less esterified with methyl groups, generated by partial acid hydrolysis or by the action of pectinase or pectate lyase (Nothnagel et al., 1983).

Fucans and carrageenans are sulfated polysaccharides present in brown and red algae whereas ulvans are heteropolysaccharides found in green algae *Ulva* spp. (Stadnik and De Freitas, 2014). The main constituents of ulvan are sulfated rhamnose residues linked to glucuronic acids, resulting in a repeated disaccharide unit β-D-glucuronosyl-(1,4)-α-L-rhamnose 3-sulfate, called aldobiouronic acid (Lahaye and Robic, 2007).

Alginates, the main extracellular matrix polysaccharides of brown algae, are constituted by poly-D-mannuronic acid (M blocks), poly-D-guluronic acid (G blocks), and alternated residues of D-mannuronic acid and D -guluronic acid (GM blocks).

### “PAMP, MAMP and DAMP type” carbohydrates

#### Perception

PAMPs, MAMPs, and DAMPs are recognized by PRR receptors (Zipfel, 2008; Macho and Zipfel, 2014). Based on the analysis of the Arabidopsis genome, the array of putative PRRs encoded in plants is much higher than in mammals. PRRs for chitin and OGAs have been identified but cognate receptors of other OS, including β-glucans, chitosan, fucan, etc., are yet unknown.

Plant PRRs are receptor-like kinases (RLKs) or receptor-like proteins (RLPs), which are localized at the plasma membrane and possess extracellular domain for ligand recognition. The major PRR types carry leucine rich repeats (LRR) or lysine motifs (LysM), while others can carry C-type lectin or EGF-like ectodomain (Shiu and Bleecker, 2003). LysM-RLKs and RLPs recognize β-1,4-linked *N*-acetylglucosamine units containing glycans and aminosugars present on microbial surface, such as fungal chitin and bacterial peptidoglycan, or lipochitooligosaccharides secreted by beneficial microorganisms (Gust et al., 2012).

In *Arabidopsis thaliana*, the chitin elicitor receptor kinase 1 (CERK1) is the key chitin binding and signaling component (Miya et al., 2007; Wan et al., 2008; Petutschnig et al., 2010). AtCERK1, a LysM-RLK with three LysM domains in the ectodomain, binds chitin directly without any requirement for interacting proteins and initiates signaling *via* its cytoplasmic Ser/Thr kinase domain (Miya et al., 2007; Wan et al., 2008; Iizasa et al., 2010; Petutschnig et al., 2010). In the monocot rice, fungal chitin is recognized by the LysM-RLP Chitin elicitor-binding protein (CEBiP) (Kaku et al., 2006; Hayafune et al., 2014; Kouzai et al., 2014). OsCEBiP is a specific chitin receptor (Kouzai et al., 2014) which cooperates with the chitin elicitor receptor kinase 1 (OsCERK1), the closest homolog of AtCERK1 in rice (Miya et al., 2007; Shimizu et al., 2010; Hayafune et al., 2014). They form a transient hetero-dimer: OsCEBiP for chitin binding and OsCERK1 for initiation of the signal transduction (Shimizu et al., 2010; Hayafune et al., 2014). In wheat, another monocot crop, homologs of CERK1 and CEBiP are both required for chitin-induced defenses (Lee et al., 2014), suggesting conserved CEBiP/CERK1 perception in monocots.

Some PRRs for DAMPs perception have also been identified. The OG receptor identified is the wall-associated kinase 1 (WAK1), a trans-membrane receptor protein kinase belonging to a family of 5 members (WAK1–5) in the Arabidopsis genome (Kohorn and Kohorn, 2012). In this gene family, only the transcripts of *WAK1* are significantly up-regulated at 1 and 3 h after OGA treatment (Denoux et al., 2008). By using a chimeric approach, Brutus et al. (2010) elegantly showed that WAK1 can bind OGAs, thus leading to the activation of its intra-membrane kinase domain to finally trigger plant immune responses. Moreover, the binding of OGA to the ectodomain of WAK1 seems to need a specific confirmation of “egg-boxes” complexes formed by calcium bridges (Decreux and Messiaen, 2005; Cabrera et al., 2008).

#### Induced defenses and resistance

From what we know, PRRs are often associated with other RLKs or RLPs to form molecular complexes (Böhm et al., 2014). Such formations can improve the ligand recognition, signal transduction or perform a regulatory role (Monaghan and Zipfel, 2012). Notably RLP receptors interact with RLKs for signal transduction. The recognition of MAMPs/DAMPs leads to the activation of the PRR kinase domain, which initiates phosphorylation and the subsequent complex cascade of signaling events that leads to defense gene activation. Defense gene expression allows the synthesis of pathogenesis-related (PR) proteins (such as the hydrolytic enzymes β-1,3-glucanases and chitinases, cationic defensin, peroxidases, proteinase inhibitors or lipid-transfer proteins), the accumulation of phytoalexins, and cell wall strengthening.

The first identified elicitor-active oligosaccharides (OS) were β-glucans produced from *Phytophthora megasperma* pv. *sojae* (Ayers et al., 1976). Thereafter, eliciting activity of OS has been largely studied (Table 1). As examples, β-1,3-glucans elicit defense responses in many species (Sharp et al., 1984; Côté and Hahn, 1994; Côté et al., 1998; Ebel, 1998; Shibuya and Minami, 2001). In particular, laminarin induces defense responses in rice (Inui et al., 1997), tobacco cell suspensions (Klarzynski et al., 2000), alfalfa (Cardinale et al., 2000) and grapevine (Aziz et al., 2003). OGAs also induce various defense responses (Ferrari et al., 2013), such as the synthesis of phytoalexins in soybean and bean (Nothnagel et al., 1983; Dixon et al., 1989), the expression of protease inhibitors in tomato leaves (Farmer et al., 1991), defense genes in Arabidopsis (Denoux et al., 2008), lignification in cucumber hypocotyls (Robertsen, 1986) or the production of active oxygen forms in many plant species (Legendre et al., 1993; Rouet-Mayer et al., 1997; Binet et al., 1998; Stennis et al., 1998; Galletti et al., 2008). Chitin derivatives induce lignification in wheat (Barber et al., 1989), ion fluxes and phosphorylation events in tomato cell suspensions (Felix et al., 1993), chitinase activity in melon (Roby et al., 1987) and the expression of glucanase in barley cells (Kaku et al., 1997). In rice, chitin triggers the MAPK cascade (Yamaguchi et al., 2013), and biosynthesis of phytoalexins and lignin (Kawano and Shimamoto, 2013).

**Figure 1:**
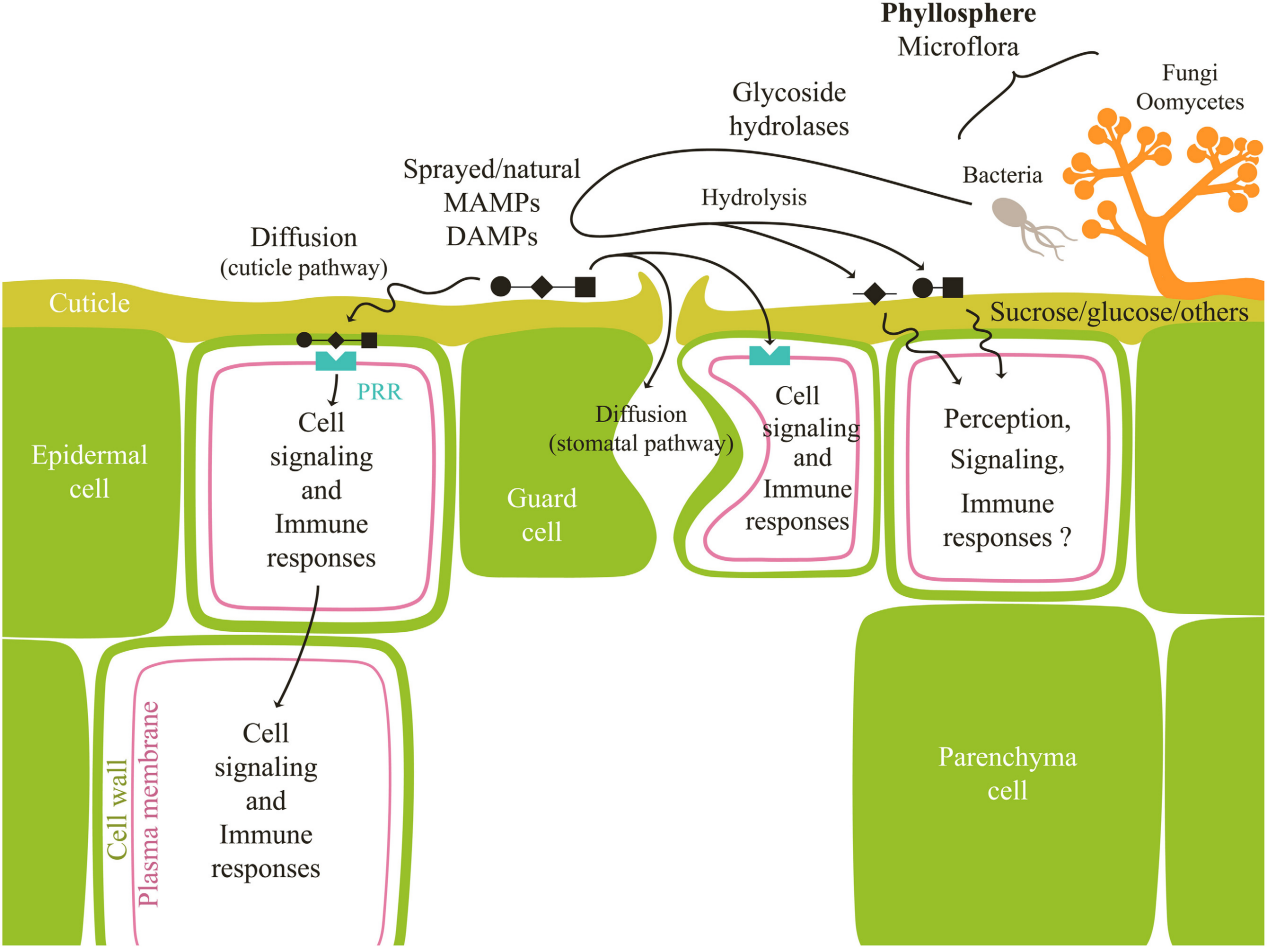
**Outcome of natural/sprayed carbohydrates at the leaf surface**. Carbohydrates have to penetrate through the hydrophobic cuticle to reach epidermal or guard cells to be perceived by PRR receptors and trigger signaling events and defense reactions (immune responses). They could also enter the leaf along the surfaces of the stomatal pores. Microorganisms living in the phyllosphere secrete enzymes susceptible to hydrolyze OS. Depending on their structure, released fragments may induce defense signaling and responses or not.

In tomato and *Commelina communis*, Lee et al. (1999) showed a H_2_O_2_-dependent induction of stomatal closure by chitosan and OGA, as in response to ABA. It was later confirmed in grapevine using β-glucans and OGA (Allègre et al., 2009) and in tobacco using β-glucans (Fu et al., 2011). These results unambiguously show that OS can be also perceived by guard cells (Figure 1), thus leading to signaling, defense activation, and also stomatal movements (generally a stomatal closure). Experiments performed with grapevine leaves also showed a higher responsiveness of guard cells to OS, compared to epidermal cells (Trouvelot et al., 2008; Allègre et al., 2009).

Numerous papers have therefore described the capacity of oligosaccharides to induce plant defenses. However, few of them have reported induced resistance of plants against pathogens. OGAs induce resistance of Arabidopsis against *Botrytis cinerea* (Aziz et al., 2004; Ferrari et al., 2007). Laminarin confers protection of grapevine against *Plasmopara viticola* and *Botrytis cinerea* and of tobacco against tobacco virus mosaic (TVM) (Klarzynski et al., 2000; Aziz et al., 2003). Treatment with chitin reduces the susceptibility of rice to *Magnaporthe oryzae* (Tanabe et al., 2006). Curiously, the effect of chitin treatment on resistance remains rather mild whereas chitosan induces a strong resistance of different plant species to fungal pathogens (Sharp, 2013). However, besides its elicitor activity (Benhamou et al., 1994), chitosan also possesses antifungal properties (El Ghaouth et al., 1994; Trotel-Aziz et al., 2006).

#### Structure/activity relationships of carbohydrates

The biological activity of oligosaccharides is highly dependent on their degree of polymerization (DP) and pattern. Fu et al. (2011) have reported that β-1,3-glucans with low DP (2–10) induce more rapid responses than laminarin with high DP (25–40) in tobacco cells. Interestingly, they observed the opposite for induced resistance: high DP β-1,3-glucans being more active than low DP ones against TVM. The highest activity of chitin was generally reported for heptamers and octamers and little or no activity for shorter oligomers (Vander et al., 1998; Hamel and Beaudoin, 2010). For chitosan, oligomers with a DP ranged between 7 and 10 are usually the most active (Hadwiger, 2013). Dissimilar size-depending biological response were also reported for OGAs (Reymond et al., 1995). DP between 10 and 16 are indeed often referred to as optimal size to induce defenses (Navazio et al., 2002; Galletti et al., 2008; Vorholter et al., 2012). For shorter OGAs with 2–6 DP, the activity is highly dependent on pathosystems. Whereas they induce defense reactions in tomato or potato (Weber et al., 1996; Simpson et al., 1998), they suppress the elicitor-induced responses in wheat leaves and hypersensitive resistance reaction in genetically resistant plants (Moerschbacher et al., 1990).

Some studies mentioned that plants may have developed the ability to react to structurally different β-glucans. In particular, soybean and rice recognize only branched β-glucans (Cheong and Hahn, 1991; Yamaguchi et al., 2000) whereas tobacco reacts to linear β-1,3-glucans. As example, the β-1,6-β-1,3-heptaglucoside elicits phytoalexin accumulation in soybean (Sharp et al., 1984) but is inactive in tobacco (Klarzynski et al., 2000).

Acetylation and methylation also impact OS activity. The chitin acetylation status is crucial for CERK1 binding as AtCERK1 can weakly bind the partially deacetylated chitosan whereas it possesses no affinity for fully deacetylated chitooligomers (Petutschnig et al., 2010). Chitosan heptaose and octaose do not elicit ROS burst and cell death in rice, suggesting that also rice requires acetylated ligands for immune activation (Kaku et al., 2006; Kishimoto et al., 2010). For OGAs, acetylation is also important since both acetylated and unacetylated ones induce defense events in wheat whereas only acetylated ones lead to an increase in papilla-associated fluorescence and to a reduction of formed fungal haustoria of *Blumeria graminis* f. sp. *tritici* (Randoux et al., 2010). The degree of methyl esterification of OGAs, modulated by pectin methylesterases (PME), is crucial for their activity (Pelloux et al., 2007). As example, a reduction of *B. cinerea* symptoms was observed in Arabidopsis plants overexpressing the specific inhibitor of PME (PMEIs) (Lionetti et al., 2007). Conversely, the overexpression of PME in strawberry leads to a reduced degree of methyl esterification of OGA, an increase of defense genes expression and an improved resistance against *B. cinerea* (Osorio et al., 2008).

Biomolecules substituted with sulfate groups are involved in major physiological functions in plants and animals. The presence of sulfate groups and the degree of sulfation (DS) can modulate the biological activities of oligosaccharides. Laminarin was therefore sulfated to improve its activity, providing PS3 with a DS of 2.4 (Ménard et al., 2004). The addition of a sulfate moiety to glucose residues in the chain is likely to modify the three-dimension structure of the molecule and consequently change its affinity to the assumed receptor (Ménard et al., 2005). Moreover, it confers PS3 resistance to degradation by endo-β-1,3-glucanases and exo-glucanases. PS3 induces increased resistance responses of tobacco infected with TMV (Ménard et al., 2004) and inhibits both virus infection and multiplication whereas laminarin inhibits only virus infection (Ménard et al., 2005), suggesting two distinct perception systems for laminarin and PS3. Moreover, PS3-enhanced resistance of grapevine herbaceous plantlets against *Plasmopara viticola* is more efficient than with laminarin (Trouvelot et al., 2008). Interestingly, PS3 acts by priming in this interaction whereas laminarin directly elicits defense events (Trouvelot et al., 2008; Gauthier et al., 2014). A structure-activity study was also conducted for laminarin sulfates having different degrees of sulfation (ranging between 0.4 and 2.4), based on PR-protein activation in tobacco (Ménard et al., 2004). A DS higher than 0.4 is required to trigger PR proteins and the activity increases with increasing DS. Moreover, the maximal eliciting activity is obtained for DS of at least 1.5 and seems to be independent of the chain length (ranging from a DP of 15 to 25 in this study).

### Sugars as signaling molecules

Sugars are also involved in plant immunity as signaling molecules (Sheen et al., 1999; Rolland et al., 2006); this has led to the “sweet-immunity” and “sugar-enhanced defense” concepts (Bolouri-Moghaddam and Van Den Ende, 2013). It concerns “small sugars” i.e., mono-, di- and small oligosaccharides (saccharide-like) such as sucrose, trehalose, raffinose or galactinol able to activate plant defense responses and increase plant resistance to pathogens. As examples, sucrose induces isoflavonoids synthesis as a defense response against *Fusarium oxysporum* in lupine (Morkunas et al., 2005). Galactinol stimulates the accumulation of defense-related gene transcripts in tobacco plants, enhances resistance against *Botrytis cinerea* and *Erwinia carotovora* and is a signaling component of the induced systemic resistance caused by *Pseudomonas chlororaphis* (Kim et al., 2008). Trehalose induces PAL and peroxidase activities associated with partial resistance of wheat against powdery mildew (Reignault et al., 2001). In Arabidopsis cell suspensions, sucrose or glucose induces the expression of several PR-genes and accumulation of the corresponding proteins PR-2 and PR-5 through a SA-dependent pathway (Thibaud et al., 2004). Conversely, sucrose, glucose, and fructose induce the PR-protein transcripts *PR-Q* and *PAR-1* in tobacco in a SA-independent pathway (Herbers et al., 1996b). As reviewed by Bolouri-Moghaddam and Van Den Ende (2012), other sugars such as psicose or D-allose can stimulate plant immunity and upregulate defense genes expression. It is tempting to think that some sugars could be considered as elicitors. However, plants may respond to changes of extracellular levels of sugars rather than sugars themselves.

How plants perceive sugars is highly complex and needs to be further investigated. The perception of hexoses is achieved by hexokinases (Granot et al., 2013; Tiessen and Padilla-Chacon, 2013) such as the intracellular glucose sensor HXK 1 of Arabidopsis (Jang et al., 1997; Sheen et al., 1999; Smeekens, 2000; Rolland et al., 2006). However, hexokinase independent pathways were also reported (Lalonde et al., 1999; Sheen et al., 1999). Sucrose and other disaccharides seem to be perceived at the level of the plasma membrane (Goddijn and Smeekens, 1998; Rolland et al., 2002, 2006; Schluepmann et al., 2003). However, sucrose can also be hydrolyzed by apoplastic invertases, providing hexoses that will be perceived by membrane or cytosolic sensors (Sheen et al., 1999). Research on sugar transporters is also in progress and will help to decipher the mechanisms associated to sugar perception.

The relationship between plant carbohydrate status and defense/resistance against pathogens has been known from a long time as the “high sugar resistance” (Horsfall and Dimond, 1957). Several papers have since reported changes in apoplastic/cell sugar content, source to sink transition, increase in cell-wall invertase activity and also changes in the sucrose/hexose ratio in plants challenged by pathogens (Bolouri-Moghaddam and Van Den Ende, 2012). Such changes are perceived by plants and allow induction of defenses (Herbers et al., 1996a; Tang et al., 1996; Ehness et al., 1997; Schaarschmidt et al., 2007; Kocal et al., 2008). Invertases, enzymes that catalyze the conversion of sucrose to glucose and fructose, are essential for the modulation of apoplastic sugar content (Roitsch et al., 2003; Roitsch and Gonzalez, 2004). Despite their role as simple soluble sugar suppliers (Bolouri-Moghaddam et al., 2010; Xiang et al., 2011), they play a key role in the regulation of source/sink relations of plants and response to pathogens (Ehness et al., 1997). This is particularly true for apoplastic invertases considered as PR-proteins (Roitsch et al., 2003). Overexpression of a yeast invertase in the apoplast of tobacco hence induces production of PR proteins and increases resistance against virus infection (Herbers et al., 2000). Transcript accumulation and/or increased activity of extracellular invertase were reported in response to glucose, sucrose, non-metabolizable sucrose analogs (Roitsch et al., 2003) and polygalacturonic acid (Godt and Roitsch, 1997). Metabolizable sugars and defense related stimuli (including chitosan) were shown to coordinately regulate source/sink relations and defense responses (Ehness et al., 1997). Such a regulation could contribute to provide energy for defenses and improve the defense response against pathogens (Roitsch et al., 2003). Interestingly, some pathogens seem to be able to bypass this signaling system. Hence, Wahl et al. (2010) have characterized the membrane–localized sucrose transporter Srt1 from the corn fungal biotrophic pathogen *Ustilago maydis*. Srt1 is sucrose specific and acts as a virulence factor as sucrose is directly taken up in the apoplast and not hydrolyzed with the subsequent release of monosaccharides able to induce defenses.

## Application of oligosaccharides for plant protection

### A still limited use

As stated above, many OS are able to induce plant defenses and, in some cases, plant resistance against pathogens in lab conditions. This has opened the way to applications in crop protection. In this context, OS are attractive candidates as resistance inducers since they are mostly non-toxic, safe to the environment and can be extracted from renewable sources. Numerous field trials with OS have been performed but few of them lead to positive and reproducible results, probably explaining why published results still remain scarce. The following part of this review presents a non-exhaustive list of experimental data representative of the present situation of OS in crop protection.

The glucan laminarin was shown to reduce severity of downy mildew and gray mold in grapevine in lab tests (Aziz et al., 2003). Unfortunately, vineyard trials yielded inconsistent results that precluded further applications for this crop. The situation is seemingly more successful with other interactions. Indeed, in field conditions, several sprays of laminarin on strawberry allowed the control of powdery mildew up to 70–80 and to 50% for leaf spot and gray mold.

Chitosan has been studied as plant protectant for more than 30 years and a wealth of publications is available. It is the OS that has given rise to the greatest number of applications (Hadwiger, 2013) in a wide array of plant-pathogen interactions and the induced protection was found to range from significant to null. For example, Iriti et al. (2011) assessed the efficacy of a commercial preparation of 85% deacetylated chitosan with a high DP (MW of 20–30 KDa) against grapevine powdery mildew. The solution was sprayed weekly at 40 mg/l during the susceptibility period of the fruits. The treatment eventually reduced the disease by more than 90% and increased the polyphenol content of berries, which suggests that this chitosan had affected the secondary metabolism of grapevine. With another commercial formulation, Sharathchandra et al. (2004) recorded up to 65% of protection against pearl millet downy mildew. According to our own experience, no efficacy of high DP chitosan preparations was observed against grapevine downy mildew in vineyard experiments (Daire, unpublished) whereas 100% protection was obtained in greenhouse conditions. Most preparations possess a strong antimicrobial activity due to the polycationic nature of the deacetylated glucosamine. Chitosan therefore has a dual role and it is often difficult to establish whether the observed protectant effect relies on the eliciting or on the antimicrobial property or both. In addition one can take advantage of biofilm forming properties of chitosan for post-harvest protection of fruits (Hadwiger, 2013).

Curiously, no published data of crop protection are available for OGAs alone while their eliciting properties have been extensively studied. Chitosan oligomers (both in the decamer range) were combined to OGA in order to stabilize the egg box conformation of the latter and the complex was shown to be a potent elicitor of defense reactions in Arabidopsis (Cabrera et al., 2010). This mixture also elicited defenses in tomato and, in greenhouse conditions, several spray applications achieved 80% protection against cucumber powdery mildew under high disease pressure. The treatment proved also effective against grapevine powdery mildew in a vineyard experiment in Spain. In this experiment, six sprays throughout the growing season, delivering only 35 g/ha of OS each, allowed to reduce disease severity from 54% in control to 13% in the treated plots (Van Aubel, 2013). Such results are in agreement with similar trials carried out previously in France (Daire, unpublished).

In spray application, oligosaccharide-induced resistance is often found to be dose dependent. For example, during greenhouse protection tests with sulfated laminarin against grape downy mildew, maximum disease reduction rate reached 85% with 5 g.l^−1^ of oligosaccharide while it was only 57% when the dose was lowered to 1.25 g.l^−1^ (Trouvelot et al., 2008). This dose-dependent effect was also observed in field trials with this OS against powdery mildew (Daire, unpublished data).

As the effectiveness of OS treatments as crop protection against diseases generally still suffers inconsistency (Delaunois et al., 2014), the role of factors susceptible to impact the level of OS-induced resistance of plants is presently investigated to develop this strategy (Walters et al., 2013). Among them are the cuticular barrier and the phyllosphere microflora.

### The cuticular barrier

Once sprayed, elicitors have to go through the cuticular barrier to reach the cell wall and plasma membrane to be perceived. The cuticle, present at the surface of plant aerial organs (Riederer and Müller, 2006) prevents water losses (Riederer and Schreiber, 2001). It is a continuous structure (0.1–10 μm thick) formed by a combination of cutin, waxes and polysaccharides (Holloway, 1982; Jeffree, 1996). Due to its chemical properties, cuticle is permeable to lipophilic compounds (Schreiber, 2006) but represents a diffusion barrier to polar ones such as carbohydrates. Polar molecules can penetrate the leaf by transcuticular hydrophilic pores, the nature of which remains unclear (Schonherr, 2006; Schreiber, 2006), or through a stomatal pathway in which substances most likely move along the surfaces of the stomatal pores (Eichert and Goldbach, 2008) (Figure 1). The cuticular pathway has rather low size exclusion limits (around 2 nm, compatible with diffusion of small carbohydrates such as sucrose) whereas the stomatal one enables entry of much greater molecules (over 43 nm in diameter) (Eichert et al., 2008). However, only a limited number of stomata seems to participate in this diffusion (less than 10% of all stomata in the case of *Allium porrum* L.; Eichert and Burkhardt, 2001). In the case of sucrose, penetration rate determined for an array of plant species was shown to range between 1% for astomatous cuticles and 4% for stomatous ones (Eichert and Goldbach, 2008). Therefore, in hypostomatal plant species, sucrose uptake across the abaxial surface was at least more than two times higher than that across the adaxial side. It is likely that penetration rate of OS, greater in size than sucrose, is even lower. These observations could account for variable and limited effectiveness of OS application as foliar sprays. For these reasons, it should be important to investigate formulation that can improve bioavailability of OS in leaf tissues (Liu et al., 2004; Fernández and Eichert, 2009).

### The phyllosphere is probably not passive regarding OS application

Leaf surfaces of nearly all higher plants form the phyllosphere (Ruinen, 1961), habitats for epiphytic microorganisms including bacteria, yeasts and fungi (Vorholt, 2012). These leaf-associated microbes use resources such as carbohydrates, amino acids, and organic acids (Tukey, 1970; Derridj, 1996; Leveau and Lindow, 2001; Van Der Wal and Leveau, 2011) passively leaked by plants. Photoassimilates like sucrose, fructose, and glucose found in abundance (0.2–2.0 μg per leaf) on uninhabited bean leaf surfaces, were indeed readily consumed and converted into biomass by the inoculated bacterium *Pseudomonas fluorescens* (Mercier and Lindow, 2000). Bacterial and fungal colonization of the phyllosphere does not occur evenly across the leaf (Kinkel et al., 1995). Hence, bacteria are more likely to be found clustered in crevices between epidermal cells (anticlinal cell walls), near the base of trichomes, in the proximity of and in stomata, and along veins (Mansvelt and Hattingh, 1987; Davis and Brlansky, 1991). This location corresponds to putative cuticular diffusion sites of hydrophilic oligosaccharides. In this context, it is pertinent to wonder about the durability of oligosaccharides once sprayed onto the leaf surface. However, bacteria of the phyllosphere secrete biosurfactants (Cooper and Zajic, 1980; Neu, 1996; Rosenberg and Ron, 1999) that increase the wettability of leaf tissues (Bunster et al., 1989; Schreiber et al., 2005) or directly alter the leaf surface permeability (Schreiber et al., 2005), so one could expect that they contribute to enhanced diffusion of xenobiotics (such as OS) through the cuticle and along stomatal pores (Eichert and Goldbach, 2008).

In another way, it is well known that microbes, especially epiphytic fungi and bacteria surviving on crop plants produce and secrete a range of enzymes, especially glycoside hydrolases that degrade cell wall polysaccharides (Culleton et al., 2013). Among them are pectinases (most notably polygalacturonases), pectin and pectate lyases and pectin esterases directed against the homogalacturonan domain, as well as rhamnogalacturonases (Alghisi and Favaron, 1995; Chen et al., 1997; Abbott and Boraston, 2008). Other microorganisms could produce chitinases and also glucanases, alginate or ulvan lyases (Lahaye et al., 1997; Da Costa et al., 2001; Urquhart and Punja, 2002; Dahiya et al., 2006). By this way, one could hypothesize different possible scenarios for a sprayed OS: *i*- it is not altered by the phyllosphere microflora, *ii*- it is partly altered and hydrolyzed by the phyllosphere microflora, resulting in a lower active quantity bioavailable for defense induction and in the release of *iii*- small fragments having a higher elicitor activity or *iv*- inactive small fragment or *v*- small sugars acting as signaling molecules (especially in the case of β-glucan) (Figure 1). Phyllosphere microflora thus undoubtedly plays a role regarding oligosaccharidic bioavailability, although it remains difficult to describe it precisely.

## Conclusion

It is now undeniable that carbohydrates play a role in plant immunity. However, their actual significance in plant-microbe interactions still remains partly unknown because of the high complexity of the mechanisms involved. As far as their use in crop protection is concerned, examples of successful applications demonstrate the potential of OS-based induced resistance as a strategy. However, OS treatments generally still suffer inconsistency. Many reasons can account for this situation among which a lack of suited formulation or degradation by epiphytic microorganisms can be hypothesized. Conversely to pesticides that act directly on pathogens, elicitor-induced resistance implies the elicitor perception by the plant and a subsequent plant response undoubtedly influenced by various factors. Progress in the identification of plant PRRs would guide the choice of the best OS candidates for crops and could be used as a criterion in plant breeding programs. PRR encoding genes could also have interest for transformation of plants lacking the corresponding PRR. The influence of various factors susceptible to modulate the plant response, such as the plant developmental stage, host and pathogen genotypes, abiotic stresses or nutrition factors, is still partially or unanswered and will require specific research. This should help OS to become part of disease control management, in combination with other strategies and reduce the use of pesticides.

## Author contributions

All authors have substantially contributed to the conception and drafting of the manuscript: Adrien Gauthier (Section Induced Defenses and Resistance), Benoît Poinssot (Sections Perception and Structure/Activity Relationships of Carbohydrates), Christelle Guillier (Section The Phyllosphere is Probably Not Passive Regarding OS Application and References), Lucie Trdá (Section Perception), Franck Paris (Sections Main Classes of Carbohydrates Involved in Plant Immunity and The Cuticular Barrier), Marielle Adrian (Introduction, Section Sugars as Signaling Molecules and supervision of drafting), Maud Combier (Section Structure/Activity Relationships of Carbohydrates and design of the figure) Marie-Claire Héloir (Abstract, Sections Main Classes of Carbohydrates Involved in Plant Immunity, Perception and Structure/Activity Relationships of Carbohydrates), Sophie Trouvelot (Sections Main Classes of Carbohydrates Involved in Plant Immunity, Structure/Activity Relationships of Carbohydrates, The Cuticular Barrier, The Phyllosphere is Probably Not Passive Regarding OS Application and Table 1), Xavier Daire (Section A Still Limited Use and Conclusion). All authors revised the manuscript critically (under the overall supervision of Marie-Claire Héloir), approved the final version to be published and agree to be accountable for all aspects of the work.

### Conflict of interest statement

The authors declare that the research was conducted in the absence of any commercial or financial relationships that could be construed as a potential conflict of interest.
